# High‐Frequency Operation of Vertical Organic Field‐Effect Transistors

**DOI:** 10.1002/advs.202201660

**Published:** 2022-06-26

**Authors:** Marco Höppner, Bahman Kheradmand‐Boroujeni, Jörn Vahland, Michael Franz Sawatzki, David Kneppe, Frank Ellinger, Hans Kleemann

**Affiliations:** ^1^ Dresden Integrated Center for Applied Physics and Photonic Materials (IAPP) Technische Universität Dresden 01069 Dresden Germany; ^2^ Chair of Circuit Design and Network Theory (CCN) Technische Universität Dresden Helmholtz Str. 18 01069 Dresden Germany; ^3^ Center for Advancing Electronics Dresden (cfaed) Technische Universität Dresden Würzburgerstr. 46 01187 Dresden Germany

**Keywords:** high‐frequency organic electronics, organic field‐effect transistors, organic thin‐film transistors, vertical transistor

## Abstract

The high‐frequency and low‐voltage operation of organic thin‐film transistors (OTFTs) is a key requirement for the commercial success of flexible electronics. Significant progress has been achieved in this regard by several research groups highlighting the potential of OTFTs to operate at several tens or even above 100 MHz. However, technology maturity, including scalability, integrability, and device reliability, is another crucial point for the semiconductor industry to bring OTFT‐based flexible electronics into mass production. These requirements are often not met by high‐frequency OTFTs reported in the literature as unconventional processes, such as shadow‐mask patterning or alignment with unrealistic tolerances for production, are used. Here, ultra‐short channel vertical organic field‐effect transistors (VOFETs) with a unity current gain cut‐off frequency (*f*_T_) up to 43.2 MHz (or 4.4 MHz V^−1^) operating below 10 V are shown. Using state‐of‐the‐art manufacturing techniques such as photolithography with reliable fabrication procedures, the integration of such devices down to the size of only 12 × 6 µm2 is shown, which is important for the adaption of this technology in high‐density circuits (e.g., display driving). The intrinsic channel transconductance is analyzed and demonstrates that the frequencies up to 430 MHz can be reached if the parasitic electrode overlap is minimized.

## Graphical abstract

The speed of operation of flexible organic thin‐film transistors is limited by the channel length. Here, ultra‐short channel vertical organic field‐effect transistors with a unity current gain cut‐off frequency (f_T_) up to 43.2 MHz operating below 10 V are shown. This also demonstrates that such devices could operate up to 430 MHz if the parasitic electrode overlap is minimized.

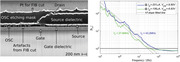

## 1 Introduction

The vision of mechanically flexible and lightweight circuits has ushered in a new era of electronics. In the future, devices such as flexible displays, wearable or even implantable sensors, for example, for health‐monitoring with a wireless communication function, and many more, are expected to accompany us in our daily lives.^[^ ^1^, ^2^ ^]^ However, severe challenges with regard to the choice of suited materials and processes to fabricate such devices need to be overcome to turn this vision into reality and avoid trade‐offs between flexibility, functionality, and performance. In particular, high charge carrier mobility semiconductor materials which fulfill the requirements for those new applications are needed to enable fast and power‐efficient circuits based on thin‐film transistors (TFT). Material classes that have been discussed in this context range from printed poly‐crystalline silicon,^[^ ^3^ ^]^ transparent conductive oxides,^[^ ^4^ ^]^ carbon nanotubes,^[^ ^5^ ^]^ graphene,^[^ ^6^ ^]^ and organic semiconductors.^[^ ^7^ ^]^ The latter class of materials is receiving a great deal of attention since such materials can be deposited virtually on any kind of substrate at low process temperatures (< 150 °C) and they offer superior mechanical flexibility in TFTs.^[^ ^8^ ^]^

Today state‐of‐the‐art organic transistors might be suited for short‐range radio‐frequency identification (RFID),^[^ ^9^ ^]^ however, they are currently not reaching the frequency standards for wireless communication, for example, in patches for mobile health‐monitoring or Internet‐of‐Things applications where data handling in frequency bands up to ultra‐high frequencies (0.3−3 GHz for medium and long‐range wireless communication) is required.^[^ ^10^, ^11^ ^]^ To bring the electrical performance, that is, the unity‐gain cut‐off frequency f_T_ of organic thin‐film transistors (OTFTs) on par with the requirements, for example, for wireless communication, improving charge carrier mobility of organic semiconductor materials has been the main thrust of research for the past 25 years.^[^ ^12^ ^]^ However, although remarkable improvements achieved by sophisticated deposition techniques have been reported, for example, record values for charge carrier mobility μ in 6,13‐bis(triisopropylsilyl‐ethynyl)pentacene (TIPS‐pentacene) of 12.3 cm^2 ^(Vs)^−1^,^[^ ^13^ ^]^ the increased mobility did not directly translate into higher cut‐off frequencies of OTFTs. The values for f_T_ employing highly crystalline and high mobility organic semiconductors are still below 38 MHz (e.g., charge carrier mobility exceeding 10 cm^2 ^(Vs)^−1^ for C_9_−DNBDT−NW as reported by Yamamura et al.^[^ ^14^ ^]^). Recently though Perinot et al.^[^ ^15^ ^]^ demonstrated record cut‐of frequency values up to160 MHz (|V_GS_| = |V_DS_| = 40 V) for solution‐processed OTFTs using a semiconducting copolymer (Poly[N,N’‐bis(2‐octyldodecyl)‐naphthalene‐1,4,5,8‐bis(dicarboximide)‐2,6‐diyl]‐alt‐5,5’‐(2,2’‐bithiophene)), which has a very low degree of structural order and hence only yields a charge carrier mobility of 0.62 cm^2 ^(Vs)^−1^. The explanation for this counter‐intuitive behavior can be found in the following correlation of the unity‐gain cut‐off frequency which is given by

(1) $$f_{T}=g_{m}/\left(2\pi C_{\mathrm{tot}}\right)=g_{m}/\left(2\pi \left(C_{\mathrm{Ch}}+C_{\mathrm{OV},\;\mathrm{tot}}\right)\right)$$

where *g*_m_ is the transconductance and *C*_tot_ is the total gate capacitance which is composed of the channel capacitance *C*_Ch_ and the total overlap capacitance between source/drain and gate *C*_OV,tot_. The transconductance is governed by a series connection of the channel resistance (inversely proportional to the charge carrier mobility and directly proportional to the channel length) and the contact resistances. For short channel length devices though (typically < 10 µm), the contact resistance becomes dominant leading to the fact that the transconductance, as well as *f*_T_, only negligibly increases if the channel length is reduced. As the contact resistance is a specific property of the semiconductor‐metal interface, device geometry, and contact engineering, the choice of low‐contact resistance materials in combination with an aggressive reduction of channel length is often the best strategy to increase *f*_T_ rather than maximizing the charge carrier mobility in long‐channel devices.^[^ ^16^ ^]^ In addition, the overlap capacitance *C*_OV,tot_ needs to be minimized by precise patterning techniques or self‐alignment^[^ ^17^ ^]^ to avoid *f*_T_ being limited by the capacitive term.

The 160 MHz device demonstrated by Perinot et al. is remarkable. However, this work also clearly shows challenges for future developments as it operates at a high bias voltage of |*V*_GS_| = |*V*_DS_| = 40 V, which is unfavorable for high‐frequency applications. Furthermore, they are fabricated on rigid, high thermally conductive substrates (AlN – 321 W (mK)^−1[^ ^18^ ^]^) to dissipate heat during operation effectively. Finally, such high frequencies of operation are reached by minimizing the parasitic overlap capacitance drastically (overlap length of ≈300 nm). However, whether such small overlap lengths can be reliably achieved in a production line on low thermally conductive substrates remains an open question.

To account for the above‐described aspects, a consensus has been found among researchers on important figures of merit for low‐voltage, high‐frequency, flexible electronics. Most important in this regard is the definition of the voltage‐normalized unity‐gain cut‐off frequency *f*_T_/V,^[^ ^19^, ^20^, ^21^ ^]^ which reflects the requirement for high frequency and low voltage operation (OTFT record value is 7 MHz V^−1[^ ^19^ ^]^ while the 160 MHz device reported in^[^ ^15^ ^]^ has a value of 4 MHz V^−1^). Furthermore, to enable power‐efficient flexible electronics, high values of *f*_T_/V need to be demonstrated on low thermally conductive substrates (e.g., flexible substrates or at least glass, the thermal conductivity of polyimide and glass are in the range of 0.1–0.8 W (mK)^−1^). Finally, high values of *f*_T_/V need to be achieved with dimensions of the electrode overlap and channel length that can be realistically employed in a low‐cost mass‐production process on flexible substrates (typically in the range of 2–5 µm).

An alternative approach to achieving sub‐µm channel length transistors for high‐frequency operation without employing high precision patterning techniques is vertical organic transistors (VOTs)^[^ ^22^ ^]^ where the current flows perpendicular to the substrate surface and the channel length is defined by the thickness of the semiconductor layer rather than a lateral patterning technique. Among the various VOTs, two designs stand out due to their performance measures: organic permeable base transistors (OPBTs) and vertical organic field‐effect transistors. As we discuss later, the vertical organic field‐effect transistors (VOFET) reported in this work is not a truly very transistor according to the upper definition as there is significant lateral transport. The OPBT, however, is a genuinely vertical device, which resembles vacuum‐tube‐triodes in which a grid‐like base electrode is employed to control the current flow from emitter to collector. The performance of state‐of‐the‐art OPBTs, which possess a vertical channel length of < 300 nm is remarkable and hero devices with *f*_T_/V = 4.65 MHz V^−1^ have been measured using a pulse‐biasing method to avoid self‐heating effects.^[^ ^23^ ^]^ Furthermore, recently Guo et al.^[^ ^24^ ^]^ demonstrated complementary ring‐oscillators based on OPBTs with a stage delay of 11 ns at a supply voltage of only 4 V as well as dual‐base devices enabling precise threshold voltage tuning for logic gates.^[^ ^25^ ^]^ However, OPBTs also have a couple of disadvantages. In particular, they are fabricated using shadow masks during ultra‐high‐vacuum deposition, which is limited in terms of the down‐scaling of devices as well as the fabrication of complex circuits. Furthermore, the function of the devices crucially relies on the thin, permeable base electrode and its fabrication is still one of the main challenges.^[^ ^26^, ^27^ ^]^

**Figure 1:**
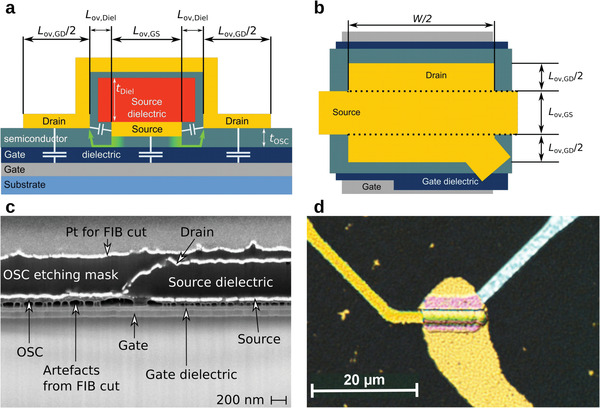
a) Sketch (cross‐section) of a VOFET illustrating the current flow and distribution of capacitances. Charge carriers are injected into the organic semiconductor (OSC) below the source electrode and reach the drain electrode by lateral diffusion and vertical drift transport. Contributions to the total capacitance of the device are indicated by capacitors (Note that the capacitance between source and drain does not contribute to *C*_tot_ in AC mode since these contacts are statically biased). b) Top view of the VOFET. The drain electrode lies flat over the source electrode. The total device area is defined by the overlapping areas of gate‐source and drain. The injection and extraction surfaces are marked with *L*_ov,GS_ and *L*_ov,GD_/2, respectively. c) Cross‐sectional scanning electron microscopy image (prepared by focused ion beam cutting, FIB cut) of a VOFET. d) Optical microscopy image of a VOFET with dimensions *W*/2 = 16 µm, *L*_ov,GD_/2 = 2 µm and *L*_ov,GS_ = 4 µm.

The second promising concept, the vertical organic field‐effect transistor, resembles a lateral transistor in its device. The gate electrode is located underneath the source electrode and not between the source and drain electrodes.^[^ ^28^, ^29^, ^30^ ^]^ The device presented in this work takes inspiration from this structure (shown in **Figure** **1**a) and hence is named VOFET. However, as discussed below, it is not a genuinely vertical transistor. The VOFET design used in this work has continuously improved in its performance measures over the years,^[^ ^31^, ^32^, ^33^ ^]^ and it can be integrated using conventional photolithography techniques. However, the dynamic properties of our VOFETs (record value *f*_T _V^−1^ = 1 MHz V^−1[^ ^33^ ^]^) are still far below the expectations for ultra‐short channel length devices. The reason for this lack of performance may be found in the fact that our VOFETs are, strictly speaking, not purely vertical. Transport occurs vertical but also to a large extent in the lateral direction (underneath the source),^[^ ^34^ ^]^ which ultimately defines the channel length and hence limits the transconductance. In consequence, the contribution of the lateral transport needs to be minimized to unravel the full potential of VOFETs and make them genuinely vertical.

Here we present a vertical organic field‐effect transistor based on the structure proposed by Kleemann et al.^[^ ^31^ ^]^ with substantial fabrication and performance improvements enabling a record cut‐off frequency of 43.2 MHz (*f*_T _V^−1^ = 4.4 MHz V^−1^) at a low driving voltage and on low thermally conductive substrates. We developed a fully scalable fabrication process based on conventional photolithography and wet/ dry chemical etching, which facilitates a compact device design and a definition of the channel length, here *L* = 200 nm, which is independent of the photolithographic resolution. We discuss the fundamental scaling laws of these vertical organic transistors and highlight the advances in their compact design over lateral TFT architectures. Although contact resistance sets the limit for the dynamic VOFET performance, we believe that the devices discussed in this work represent a major breakthrough for the development of high‐frequency organic transistors.

## 2 Results and Discussion

In the following, we describe the geometrical aspects that govern the VOFET operation and performance. Based on this understanding, we discuss the DC‐operation for different geometries and semiconductor materials. Finally, we analyze the AC‐performance of the optimized VOFETs and discuss the scaling laws connecting the device geometry and dynamic properties.

### 2.1 Fabrication, Structure, and DC Properties

The cross‐sectional scheme and top‐view of a VOFET are displayed in Figure 1a,b. Charges are injected from the central source electrode (*S*) into the organic semiconductor (OSC) due to an applied gate‐source voltage *V*_GS_ and accumulate at the interface between OSC and gate dielectric.^[^ ^34^ ^]^ With an applied drain‐source voltage *V*_DS_, a vertical electric field is established and charge carriers are drawn from the edges of the source electrode. The lateral transport of mobile charges along the gate dielectric is established by diffusion. To suppress direct current between source and drain (*D*) electrode, a dielectric material (denoted here as source dielectric with thickness *t*_Diel_ with a defined overlap *L*_ov,Diel_ is introduced on top of the source electrode. This layer allows for a sufficiently low off‐current since no direct path between *S* and *D* along the applied electric field is given and charges are forced to flow by lateral diffusion before vertical drift transport towards the drain electrode sets in.^[^ ^34^ ^]^ The length of the 2D current path, which can be seen as equivalent to the channel length *L*, is composed of the length of the lateral overlap of the source dielectric *L*_ov,Diel_ and the vertical thickness of the organic semiconductor layer. The layer thickness of the organic semiconductor can easily be scaled down to below 25 nm via the amount of deposited organic material and hence enable very high current densities. However, the crucial parameter to maximize the current within the device is minimizing the overlap of the source dielectric *L*_ov,Diel_ while still maintaining its capability to block direct charge flow from source to drain. For this purpose, various techniques for lateral structuring have been developed, such as evaporation through shadow masks,^[^ ^35^ ^]^ lift‐off,^[^ ^31^ ^]^ or orthogonal double‐resist lithography.^[^ ^32^, ^34^ ^]^ However, these methods have clear disadvantages such as blurring of edges for shadow mask patterning or the need for a separate alignment step for double‐resist lithography. A lift‐off process for the source metal and an inorganic source dielectric as utilized in^[^ ^31^ ^]^ is also not favorable since the material at the edge of the source and dielectric experiences mechanical strain and thus produces an inhomogeneous edge, which makes the crucial depth of the undercut L_ov,Diel_ hard to control.

Here we are employing a novel procedure to generate ultra‐short overlaps down to the sub‐µm regime (see Supporting Information for details on the fabrication process and Table S1, Supporting Information for details on the organic semiconductor materials being used in this work): The excess source metal is removed in a wet etching process which is carried out directly on the organic semiconductor. At the same time, the photoresist on top of the source electrode is employed as the source dielectric material providing proper electrical insulation up to voltages of 20 V (thickness of the source insulator is 400 nm). The depth of the underetch defining *L*_ov,Diel_ is determined by the concentration and type of the etchant and the etching time, and it can be adjusted well during the fabrication process (the control of the etching process is shown in Figure S1, Supporting Information). In particular, there is a window of ≈30 s during the etching process where the structure appears to be completed etched but not yet underetched in the optical microscope. This time window can even be increased by further diluting the etchant. For the chosen etching time of 95 s, the underetch (*L*_ov,Diel_) of the devices analyzed in this work is as low as 200 nm (see Figure 1c). Employing this process, we integrate the VOFETs with varying device dimensions (*W*/2, *L*_ov,GS_, *L*_ov,GD_ /2, cf. Figure 1b) into a ground‐source‐ground electrode configuration for electrical characterization (see Figure 1d).

**Figure 2:**
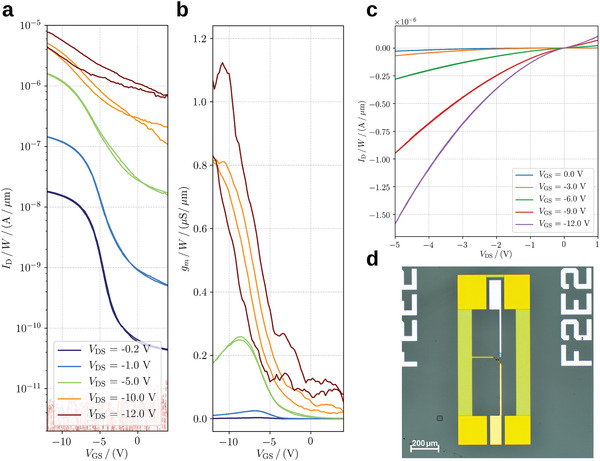
a) Transfer curve and b) transconductance of a VOFET with DNTT as OSC and undoped contacts (*W*/2 = 12 µm, *L*_ov,GS_ = 6 µm, *L*_ov,GD_/2 = 4 µm). c) Output characteristics of the same device under forwarding bias conditions (source electrode is kept at ground potential). d) A ground source ground (GSG) structure is used for the S‐Parameter measurement.

In **Figure** **2**a,b we show a typical transfer curve of a DNTT‐based VOFET with a channel width of *W*/2 = 12 µm for voltages up to *V*_DS_ = −20 V and *V*_GS_ = −12 V. These devices show almost no hysteresis and channel‐width‐normalized transconductance of up to 1.1 S µm^−1^. The instability at higher voltage is caused by the soft breakdown of the source dielectric material of this specific device. This effect statistically occurs in the voltage range between *V*_DS_ = −12 V… −16 V. We intentionally have chosen to show a device with this instability at V_DS_ = −12 V to make the reader aware of this effect. The voltage range can be increased by using thicker dielectric materials on the source electrode. However, for a thickness of > 700 nm, the drain electrode might cover the step incompletely, bearing the risk of losing contact. Besides the high transconductance, the devices show an increasing off‐state current with increasing drain‐source voltage, which is a problem frequently observed in short‐channel transistors.^[^ ^15^ ^]^ We hypothesize that the increasing off‐state current is caused by unintentional p‐type doping of DNTT due to oxygen. Hence, due to the increased background density of free charge carriers, the channel cannot be fully depleted at high *V*_DS_ anymore causing a linear increase of the off‐state current.^[^ ^36^ ^]^ As can be seen in Figure S2, Supporting Information, this effect is significantly reduced if DPh–BDT is used as semiconductor material due to its deep highest occupied molecular orbital preventing its oxidation in the ambient. In addition, the increasing off‐state current for VOFETs might be caused by the size of the channel width *W*/2 < 20 µm. In particular, parasitic current flow via non‐gated areas is unavoidable in such small devices with minimized overlap capacitances. Overall, the poor on/off ratio of DNTT‐based VOFETs is a severe problem for some applications (e.g., the switching TFT in an active matrix display). However, the on/off ratio of DNTT‐based VOFETs might be sufficient for fast amplifiers or fast digital circuits. In addition, the good on/off switching of DPh‐DBT VOFETs provides a guideline for the development of new semiconductor materials allowing for fast operation and good switching properties.

The output characteristics of a DNTT‐based VOFET are shown in Figure 2c. These devices do neither show a distinct linear nor saturation regime as usually seen for long‐channel OTFTs. Instead, the shape of the output curve resembles the current‐voltage curve of a diode. Such behavior might be either caused by the short‐channel effects (e.g., drain‐induced barrier lowering, etc.) or by the presence of a large, voltage‐dependent contract resistance. Taking best values from the literature for the contact resistance in staggered bottom‐gate OTFTs with DNTT as semiconductor (≈100 Ωcm,^[^ ^16^ ^]^), we conclude from estimating the total device resistance from the output curve that the DNTT‐based VOFETs are only limited by the charge carrier injection with the value of the contact resistance strongly depending on the gate‐source and drain‐source voltage (see Supporting Information for more information about the estimation of contact resistance). This hypothesis is confirmed by analyzing the output characteristics for swapped electrodes (see Figure S3, Supporting Information). As the semiconductor below the upper electrode (now source rather than drain) has been exposed to an etchant for structuring the lower electrode before the deposition of the metal layer, charge injection is expected to be further impeded. As seen in Figure S3, Supporting Information, the current is significantly reduced if the electrodes are swapped, which is consistent with the expectation that the contact resistance is even higher.

This strong impact of contact resistance is even more severe in VOFETs using DPh‐DBT as semiconductor material (see Figure S4a,b, Supporting Information). Due to its high ionization potential (see Table S1, Supporting Information), the height of the contact barrier to the source electrode material is expected to be even higher than in the case of DNTT ultimately resulting in higher contact resistance. Assuming that the current in the VOFET is only limited by the contact resistance, we estimate the value of the contact resistance from the output characteristics under forwarding bias conditions to be 2–4 kΩcm. In this context, it is worth noting that typical methods to determine the contact resistance based on a variation of the channel length cannot be applied to VOFETs due to their specific design.

To improve the transport and lower the injection barrier, we evaluate the use of molecular doping underneath the source electrode. Before the deposition of the source electrode material, we deposit 2 nm of the strong acceptor molecule F_6_TCNNQ onto the layer of DPh–BDT. As shown in Figure S4c,d, Supporting Information, the current significantly increases compared to the undoped devices, and we can provide a lower estimate for the contact resistance of Au/F_6_TCNNQ/DPh‐DBT from the output characteristics to be < 1 kΩcm. We also evaluated the strategy of contact doping for DNTT‐based VOFETs where we have seen a similar increase in on‐state current. However, the presence of the dopant in the semiconductor layer further increases the concentration of background charge carriers in DNTT, which leads to the fact that the transistor can hardly be switched off. Hence, we think that contact doping is not a workable approach to reducing contact resistance for DNTT‐bases VOFETs.

Another important device design parameter to optimize the charge injection in the staggered transistor configuration is the width of the source/drain electrode defining the area of overlap to the gate which is available for charge carrier injection/ extraction. As we aim for high‐frequency operation, the width of the source/drain electrode needs to be small to reduce the device capacitance but it should be large enough to not limit the charge carrier injection /extraction. For this purpose, we fabricated devices with different widths of the drain electrode and compare the on‐state current, which gives a good indication of the strength of the contact resistance. As shown in Figure S5, Supporting Information, we observe that for devices with *L*_ov, GD_ = 2 µm and *L*_ov, GD_ = 4 µm there is a significant difference in the on‐state current for small values of *V*_DS_ = −0.2 V. However, at *V*_DS_ = −5 V, we do not obtain any difference in the on‐state current for the different devices from which we conclude that the drain‐source field improves the charge carrier injection in such short‐channel transistors. Hence, the transconductance is not limited by the size *L*_ov, GD_ at high *V*_DS_ for the electrode dimensions that can be fabricated by the photolithography process proposed here (metal line width typical ≈2 µm).

### 2.2 AC‐Properties and Device Scaling

The unity‐gain cut‐off frequency as derived from the small‐signal current gain |h_21_| is the most important figure of merit characterizing the AC‐performance of a transistor. The biggest challenge to reaching high values of *f*_T_ is to maximize the transconductance *g*_m_ at a low driving voltage and simultaneously minimize overlap capacitances (cf. Equation (1)). Therefore, not only the properties of the organic semiconductor such as charge carrier mobility, charge carrier injection, and extraction properties are crucial but also the device geometry which determines the capacitance. Using sophisticated alignment methods and devices with a large channel width *W* compare to the channel length *L*, the overlap capacitance and the contribution of parasitic capacitances at the edges of the device can be reduced significantly. However, in this regard, two aspects should be kept in mind. First, alignment and patterning techniques that can be used in a low‐cost production process on flexible substrates are restricted to a resolution in the range of a few micrometers, and second, the areal footprint of the device should be kept small in order to allow for high‐density integrated circuits (e.g., active matrix displays) which excludes the use of devices with very wide channels (W < 100 µm). For this reason, we adapt conventional photolithography as an established and low‐ to medium‐cost patterning technique and we restrict ourselves to small areal footprint devices. In particular, we investigate devices with a footprint as small as 12 × 6 µm^2^ for the analysis of the cut‐off frequency.

One specific advantage of the VOFET design over horizontal OTFTs is their small areal footprint and hence small device capacitance. In Figure S5, Supporting Information, we compare the device area and total gate capacitance for a VOFET and OTFTs for design tolerances of the electrode overlap. As shown in Figure S5, Supporting Information, the VOFET has only half of the device capacitance of an OTFT with similar design rules (*L* = *L*_OV,GD_ = *L*_OV,GS_ = 2 µm) for a channel width of > 100 µm. This advantage of the VOFET is getting less for devices with smaller W (due to unavoidable alignment tolerances) but still remains significant for devices with *W*/2 = 10 µm. Furthermore, it should be kept in mind that the VOFET will provide a higher on‐state current than the OTFT due to its short channel length. Overall, the VOFET design is expected to be superior due to the reduced capacitance and the high transconductance.

**Figure 3. a):**
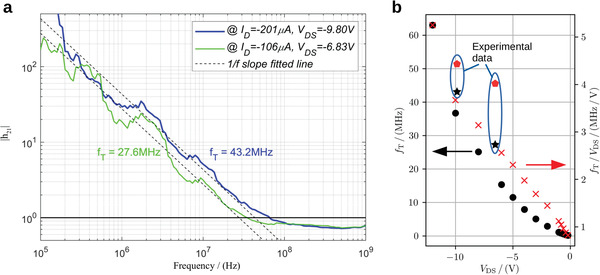
Small‐signal performance of the VOFET extracted from measured S‐parameters as current gain *h*_21_ and cut‐off frequency *f*_T_ at two bias points. b) Cut‐off frequency versus applied *V*_DS_, calculated from the shown DC transconductances at their maximum value.

To evaluate the AC properties of the VOFETs, we carry out S‐Parameter measurements. For that purpose, the VOFETs are continuously biased and are stable at the DC operating point for > 40 s (see Supporting Information for details). We determine the small‐signal gain |h_21_| as a function of the frequency. As shown in **Figure** **3**a, the VOFET has a unity‐gain cut‐off frequency of 27.6 and 43.2 MHz at *V*_DS_ = −6.83 V and *V*_DS_ = −9.80 V, respectively, which translates into voltage‐normalized values of 4.1 and 4.4 MHz V^−1^ (see Figure 3a). These are the highest values reported for organic transistors integrated by scalable methods such as photolithography or printing. We expect a cut‐off frequency of up to 62 MHz (5.2 MHz V^−1^) from a prediction based on the DC characterization of *g*_m_ and the small‐signal capacitance (see Figure 3b). However, some devices do not operate reliably at higher *V*_DS_ and hence, we excluded measurements at higher *V*_DS_ from this experimental analysis. Still, this calculation is probably even underestimating the true cut‐off frequency as it does not include self‐heating effects.^[^ ^23^ ^]^ Note that the measurement of h_21_ shown in Figure 3a is noisy, which is due to the small capacitance of these devices (*C*_tot_ = 127 fF). However, the capacitance extracted from the AC analysis matched with an error of < 15% with the value extracted from the device area and specific dielectric capacitance (*C*_tot,A_ = 107 fF). This fact confirms that the noise (and possibly stray capacitances) do not influence the AC analysis (see Supporting Information for more details on the AC analysis). Moreover, we would like to point out that the 43.2 MHz device is not a hero device but rather represent the typical performance of these devices (see Supporting Information for details).

To provide a perspective on the possible limits of VOFETs, we show in Figure S6, Supporting Information the transconductance of the active VOFET channel as a function of frequency at the same bias condition as used in Figure 3a to reach *f*_T_ = 43.2 MHz. The transconductance is the most important small‐signal parameter of the transistor, and for DC operation it is defined as *g*_m_ = *dI*_D_/*dV*_GS_|_VDS = const_. In long channel transistors, this quantity can be calculated as *g*_m_ = *μ C W/L* (*V*_GS_ ‐*V*_th_) with *C* being the specific dielectric capacitance of the gate dielectric and *V*_th_ being the threshold voltage. Since *g*_m_ is proportional to *C*, any frequency dependence of the gate dielectric constant, *ε*_r_, would result in a similar frequency dependence of *g*_m_. This effect is, for example, confirmed in the planar OTFT measurements.^[^ ^37^ ^]^ In an ideal transistor, any change of *V*_GS_ would result in an immediate change of the *I*_D_, that is, g_m_ is a positive real number with no complex part. A real transistor channel, however, is a distributed R–C network, and redistribution of electric charges in the channel in response to gate‐source voltage takes time. This effect also causes a loss of g_m_ and g_m_ becoming a complex number at high frequencies. The negative imaginary part of *g*_m_ means an additional delay in the circuit response from input to output and therefore is not desirable. The small‐signal model of the transistor is shown in the subset in Figure S6, Supporting Information, and g_m_ can be calculated as *g*_m_ = *Y*_21_ – *Y*_12_ from Y‐parameters which are extracted from measured S‐parameters. The useful, real part of g_m_ shows excellent stability and < 1 dB loss up to 430 MHz. This finding confirms the operational stability of the dielectric layers at high frequencies and means that the VOFET could amplify signals at such frequencies if we could reduce the overlap capacitance *c*_gs_ and *c*_gd_ further. In other words, there is a big room to further improve this organic transistor to operational frequencies of several hundreds of MHz.

## 3 Conclusion

In summary, we presented vertical organic field‐effect transistors with a very low areal footprint (*W*/2 = 12 µm) which stably operate above 43.2 MHz (4.4 MHz V^−1^) on low thermally conductive substrates. These devices have been fabricated using conventional photolithography and wet/ dry etching processes which can be seamlessly adapted in a low‐to‐medium‐cost mass production environment. The VOFET design offers significant benefits over lateral OTFTs in terms of capacitance reduction which might be the key to enabling operation over 100 MHz. Furthermore, our work shows that even with materials such as DNTT, which possess moderate charge carrier mobility in the range of 1 cm^2 ^(Vs)^−1^, a high‐frequency operation is possible in ultra‐short channel length vertical organic transistors. In particular, other material properties such as intrinsic background charge carrier density (e.g., due to oxygen‐induced doping or light) or the injection resistance should get into the focus on the development of new organic semiconductor materials to enable organic electronics operating in the GHz‐regime. Frequencies up to 430 MHz could be reached with the set of materials if the parasitic electrode overlap is minimized.

## Conflict of Interest

The authors declare no conflict of interest.

## Supporting information

Supporting Information

## Data Availability

The data that support the findings of this study are available from the corresponding author upon reasonable request.
