# Supplementary material for: High‐Frequency Operation of Vertical Organic Field‐Effect Transistors
Source: Adv Sci (Weinh). 2022 Jun 26;9(24):2201660. doi: 10.1002/advs.202201660 (PMC9403633; doi:10.1002/advs.202201660)
Supplement: Supplementary file 1 — Supporting Information [file ADVS-9-2201660-s001.pdf]

Supporting Information

High-Frequency Operation of Vertical Organic Field-EffectTransistors

M. Höppner et al.

Sample preparation: Glass substrates are cleaned in an ultrasonic bath with isopropanol and acetone for 5 minutes each and subsequently exposed to oxygen plasma for 5 minutes at 200 W. To produce the gate electrode, a 40 nm thick layer of aluminium is deposited onto the substrates via physical vapor deposition (PVD) at a base pressure of 10^{-7} mbar and a constant rate of 0.05 nm/s which is controlled with a quartz crystal monitor. The lateral patterning is established with a first photolithography step in which with 1-methoxy-2-propanol acetate (PGMEA) diluted photoresist AZ nLof 2020 by MicroChemicals GmbH (PGMEA : nLof 2020 - 3:2 vol.%) is used. In ambient air, the resist is spin-coated onto the substrate with 3000 rpm, pre-baked at 110°C on a hotplate for 2 minutes, exposed with the mask aligner tool MJB4 (SÜSS MicroTec) through a pre-patterned lithography mask (Compugraphics Jena GmbH) with a dose of 40 mJ/cm^2 and post-baked on a hotplate at 110°C for 2 minutes. For developing the resist, the substrate is dipped into the tetraethylammonium hydroxide (TMAH) based developer maD 533s (Micro Resist Technology GmbH) for 15 seconds. Excess aluminium is then removed by etching for 1 minute in TechniEtch Al80 by MicroChemicals GmbH while the gate structures are protected by the patterned resist. The remaining resist material is removed with acetone in a ultrasonic bath for 1 hour and subsequent oxygen plasma treatment for 15 minutes at 200 Watt. The gate dielectric of 35 nm thick Al_2O_3 (dielectric constant 7.8) is made by atomic layer deposition (ALD) and structured with an overlap around the area of the the gate electrode. The same photolithography step as for the gate electrode is utilized to structure the oxide. The removal of the oxide takes place due to

the direct contact of the Al_2O_3 with the developer maD 533s. The second layer of the gate-dielectric is established by the deposition of 40 nm fluoropolymer Cytop^{TM} (AGC Chemicals) diluted in Cytop^{TM} Solvent CT 180 - 2:7 vol.% by spin-coating at 3000 rpm with subsequent heating on a hotplate at 100°C for 5 minutes and 150°C for 5 minutes. The specific capacitance of the dielectric layers (Al_2O_3 and Cytop^{TM}) is 50nF/cm^2. In the following, 25 nm of the organic semiconductor material and if applicable 2 nm of the dopant are deposited by PVD followed by a 40 nm thick layer of gold to build the source electrode. The gold is then treated in lithography step analogously to the first whereby standard gold etchant by Sigma-Aldrich diluted in distilled water (etchant : water - 1:10 vol.%) is used to remove the excess gold. The remaining nLof resist is not stripped afterwards but remains on the source electrode and serves as source dielectric with a thickness of 400 nm. In the following, a second, 25 nm thick layer of the OSC and if applicable 2 nm of dopant are deposited. To establish the drain electrode, 40 nm of gold are deposited via PVD. The lateral structuring for the drain electrode is carried out employing fluoropolymer-based photolithography which is benign to organic semiconductors [48]. The photoresist OSCOR 5001 (Orthogonal, Inc.) is used which is spincoated onto the sample with 6000 rpm, pre-baked at 100°C on a hotplate for 1 minute, exposed by the mask aligner tool through a pre-patterned lithography mask with a dose of 30 mJ/cm^2, post-baked at 100°C and subsequently developed with Developer 100 (Orthogonal Inc.). An alignment accuracy of ~ 2 µm is reached. The excessive gold is etched in the same manner as for the source electrode. Remaining OSCOR 5001 is removed with OSCOR Stripper 903. In a last lithography step, the OSC is laterally structured. OSCOR 5001 is used in a similar fashion as for the drain electrode. However, here it is spin-coated with 2000 rpm to obtain a thicker layer and the exposure dose after pre-bake is 70 mJ/cm^2. The post-bake is at 120°C for 2 minutes after development. Subsequently the sample is exposed to oxygen plasma (Atto plasma cleaner by Diener) at 200 W with an O_2-flowrate of 50 sccm and process pressure of 4 mbar for 30 seconds in the case of DPh-BDT and 45 seconds for DNTT. The

samples are exposed to ambient air in between every production step including the steps which followed the PVD of the OSC and measurements were performed in air, too.

Measurements: S-parameters are measured using a Rohde&Schwarz ZVL6 Network Analyzer, and then converted to the h-parameters and Y-parameters using MATLAB s2y and s2h functions. In the overlap area between the gate and drain we have a stack of dielectric layers and organic semiconductor. However, in Figure S6 whatever the electrical admittance between the gate and drain (y_{gd}) is, we would have $Y_{21} = g_m - y_{gd}$ and $Y_{12} = y_{gd}$, therefore we always have $g_m = Y_{21} – Y_{12}$.

The measurement of h_{21} takes at least 40s. It is simply the time that our Network Analyzer requires to measure the S-parameters at 121 frequency points. Since the frequency of measurement is <1GHz and the input/output capacitances of the VOFET are quite small, <<1pF, the small-signal currents flowing into the gate and drain terminals are very small. For this reason, we had to configure the Network Analyzer on very accurate settings and this makes the measurement so slow. The device is continuously operated during this time at the relevant bias point. In this way, we ensure that the device has stabilized, i.e., heating effects, etc., can be neglected [23]. The devices can be operated under such conditions for even longer durations. However, it should be noted that, as our devices are not encapsulated), degradation might still occur on the time scale of days and weeks (e.g., due to degradation of DNTT [38]). However, it has been shown previously that vertical transistors may show similar long-term stability as horizontal OTFTs or other TFT technologies [33, 49].

As we have intentionally chosen small footprint devices with small capacitance, the measure of h_{21} is noisy due to the small displacement currents at low frequency. However, from the linear trend of h_{21} vs frequency and the transconductance (gm=34.1µS), the device capacitance can be extracted (C_{tot}= 126 fF) and it agrees with an error <15% with the value estimated from the device area and specific oxide capacitance ($C_{tot,A}$= 107fF). Furthermore,

the constant h_{21} value reached at high frequencies above f_T, where the transconductance current becomes smaller than the capacitive currents, can be calculated to reach the limit of $C_{gd}/(C_{gs}+C_{gd})$. In this specific sample we had $L_{OV,GS}=L_{OV,GD}/2$, therefore this value shall be around 2/3 which is in agreement with the measurement..

Estimation of contact resistance: We estimate the contact resistance based on the assumption that the device is not limited by the conductivity of the vertical channel but rather only by the contact resistance (similar to the transmission line method where the contact resistance is extracted for channel length equals zero). In this context, contact resistance includes the injection resistance at the metal-semiconductor contact and the layer underneath the source electrode, where transport is mainly governed by diffusion rather than drift (drift transport only occurs in the vertical direction).

Due to this strong contact resistance limitation, the devices do not show a linear output curve (e.g., in Fig. S3). However, the contact resistance can be roughly estimated by linearizing these curves. For example, at V_{GS}=-12V, we obtain a resistance of ~200Ohm cm (between V_{DS}=-1.5V and -5V), assuming that this entire resistance is due to contact resistance. This value is very close to values reported in the literature (~100Ohm cm for DNTT Au [16]), and hence, we conclude that our assumption of the dominance of contact resistance is correct.

Device yield and reliability of operation: The operation of VOFETs is reproducible and generally also uniform. Typically, the operation yield is in the range of 90% for devices with W>100 [33]. For small devices, the yield may go down due to the mechanical instability of electrodes (e.g., due to dust particles). The uniformity of operation is shown below for devices with W>100. It is usually around 10-20% for the on-state current for small devices.

Also the yield of operation of devices used for the AC analysis is high. However, we cannot quantify the yield and variable of f_T as we have only two devices on this mask layout with identical geometries. However, the device reported with 43.2 MHz is not a hero device. In fact, device with W>100 µm operate even faster due to the reduced influence of overlap capacitances.

Structure	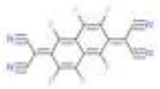	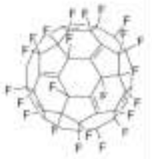	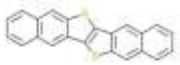	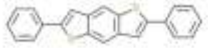
Short name	F_6TCNNQ	$C_{60}F_{36}$	DNTT	DPh-BDT
IUPAC name	1,3,4,5,7,8-hexafluoro-tetracyanonaphtho-quinodimethane	$C_{60}F_{36}$	Dinaphtho (2,3-b:2',3'-f)thieno-(3,2-b)thiophene	2,6-diphenylbenzo-(1,2-b:4,5-b')thiophene
Mobility [cm^2/(Vs)]	-	-	0.8 - 3.0 [38-40]	4.6 x 10^{-3} [41]
Electron affinity [eV]	-5.37 - -5.60 [42, 43]	-5.38 [44]	-2.10 [45, 46]	-2.04 [47]
Ionization potential [eV]	-7.53 [43]	-8.38 [44]	-5.2 - -5.44 [45, 46]	-5.50 [47]

Table S1: Organic semiconductor materials being used in this work with a summary of their most important properties (References given for properties refer to the list of references from the main manuscript).

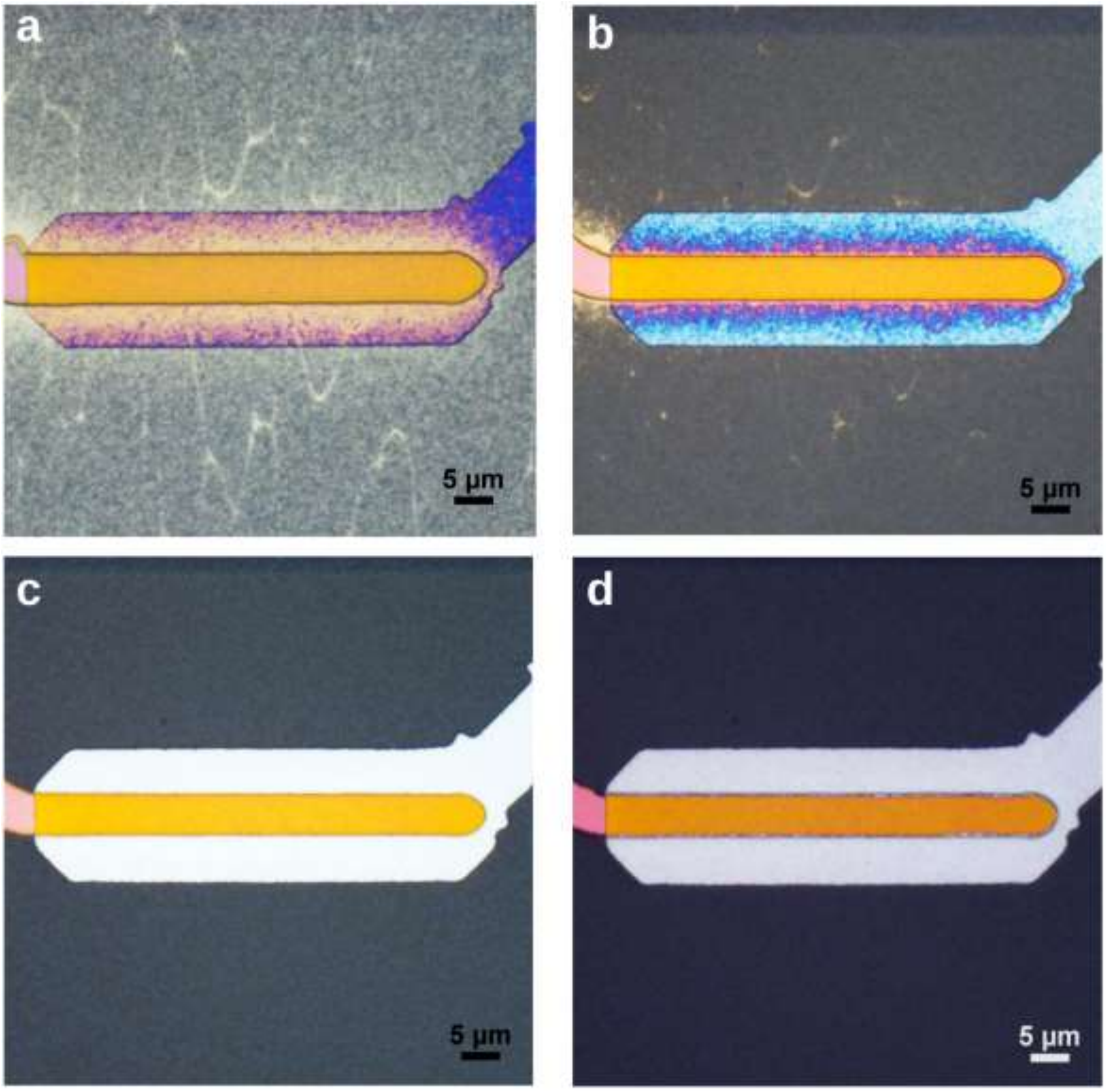

Figure S1: Microscope images of a typical VOFET source electrode etching process as described above with W/2 = 50 µm, $L_{ov,GS}$ = 4 µm, $L_{ov,GD}/2$ = 4 µm **a** after 30 seconds, **b** 55 seconds, **c** 95 seconds, and **d** 130 seconds of etching time.

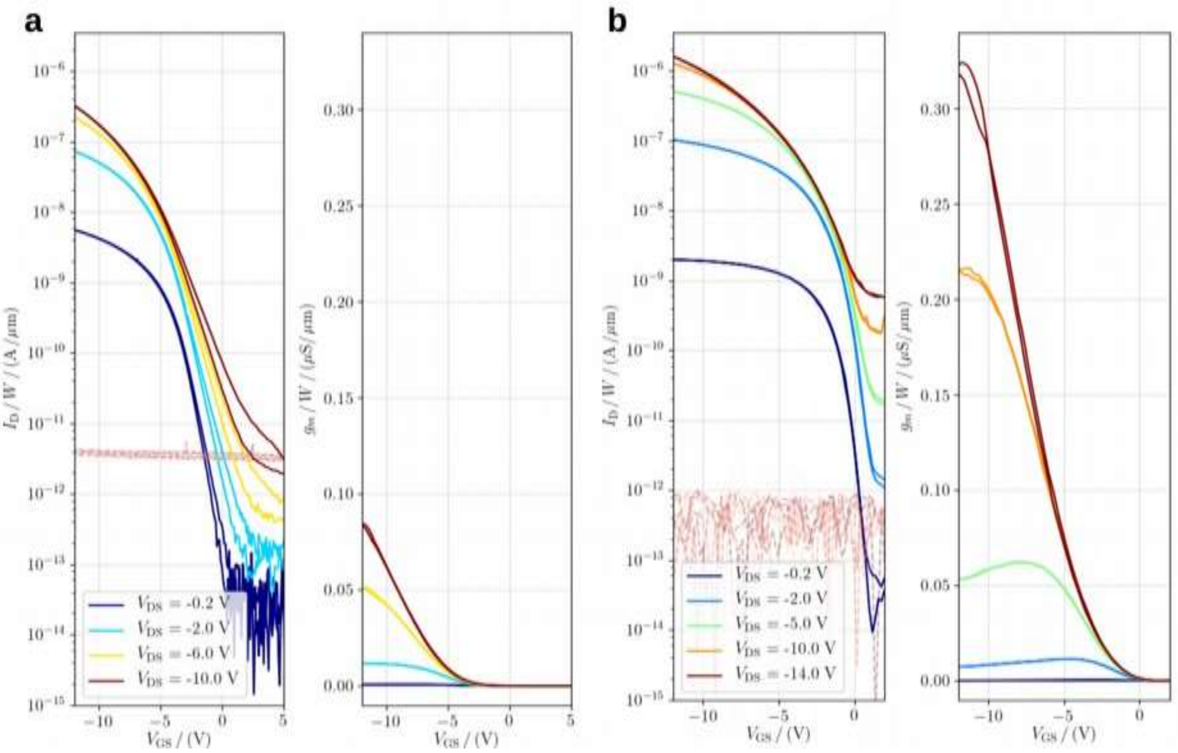

Figure S2: Transfer and transconductance of a VOFET with DPh-BDT as OSC. **a** Undoped VOFET with DPh-BDT as OSC (W/2 =25 µm, $L_{ov,GS}$ = 4 µm, $L_{ov,GD}/2$ = 4 µm), and **b** source contact doped with 2 nm F_6TCNNQ (W/2 = 50 µm, $L_{ov,GS}$ = 6 µm, $L_{ov,GD}/2$ = 4 µm).

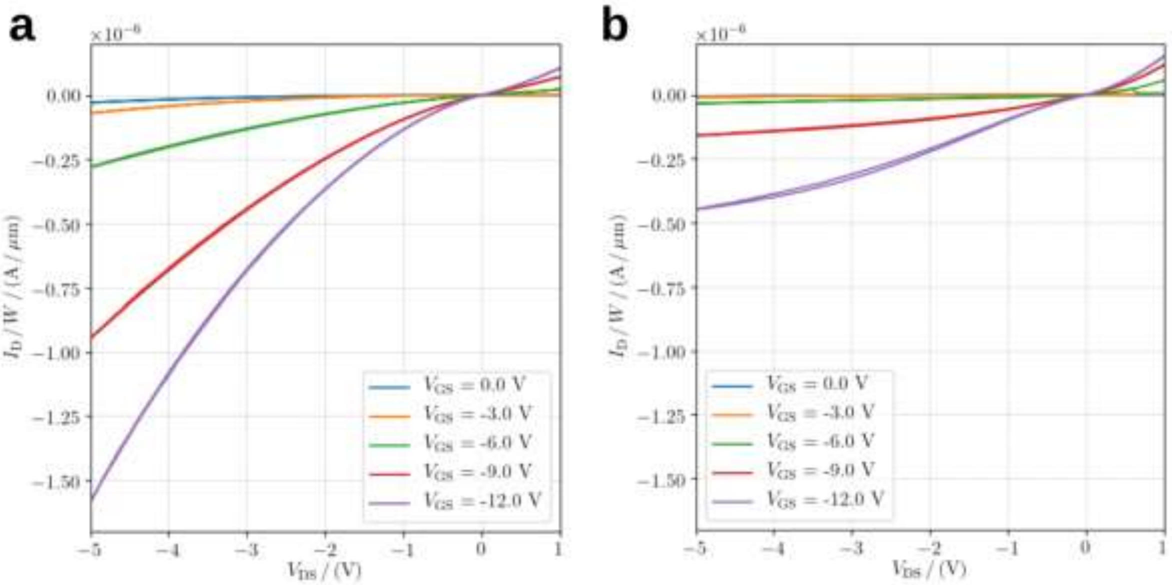

Figure S3: Output characteristics of the VOFET shown in Figure 2 with undoped DNTT as OSC (W/2 = 12 µm, $L_{ov,GS}$ = 6 µm, $L_{ov,GD}$/2 = 4 µm) under **a** forward bias conditions (lower electrode is used as source electrode and kept at ground potential 0 V) and **b** reverse bias conditions (upper electrode is used as source electrode and kept at ground potential).

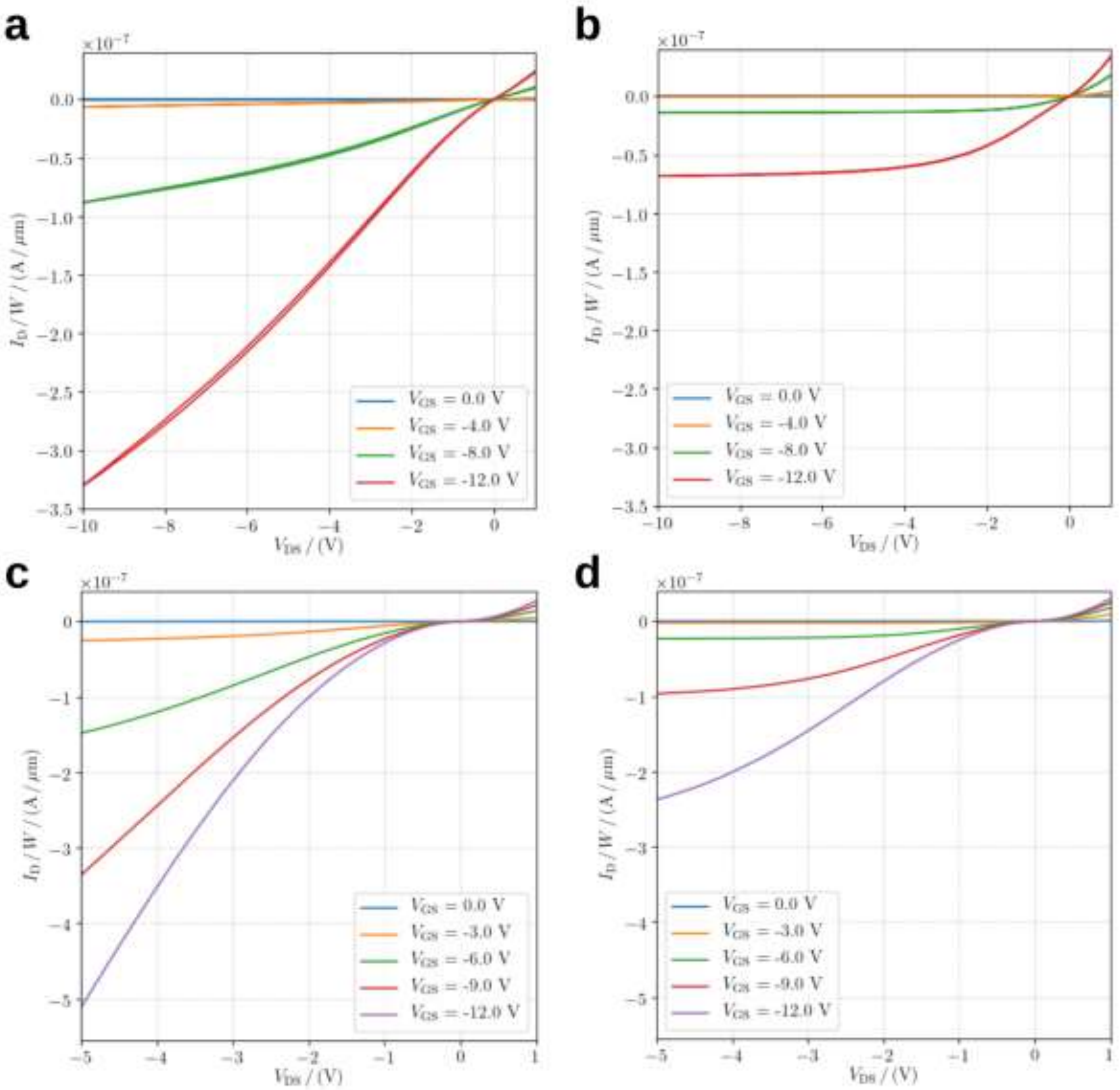

Figure S4: Output characteristics of a VOFET with undoped DPh-BDT as OSC (W/2= 50 μm, $L_{ov,GS}$= 4 μm, $L_{ov,GD}$/2 = 2 μm) under **a** forward bias conditions (lower electrode is used as source electrode and kept at ground potential 0 V) and **b** reverse bias conditions (upper electrode is used as source electrode and kept at ground potential 0 V). Output characteristics of a VOFET with DPh-BDT as OSC and contact doping with 2 nm F_6TCNNQ (W/2 = 50 μm, $L_{ov,GS}$ = 6 μm, $L_{ov,GD}$ /2 = 2 μm) under **c** forward and **d** reverse bias conditions.

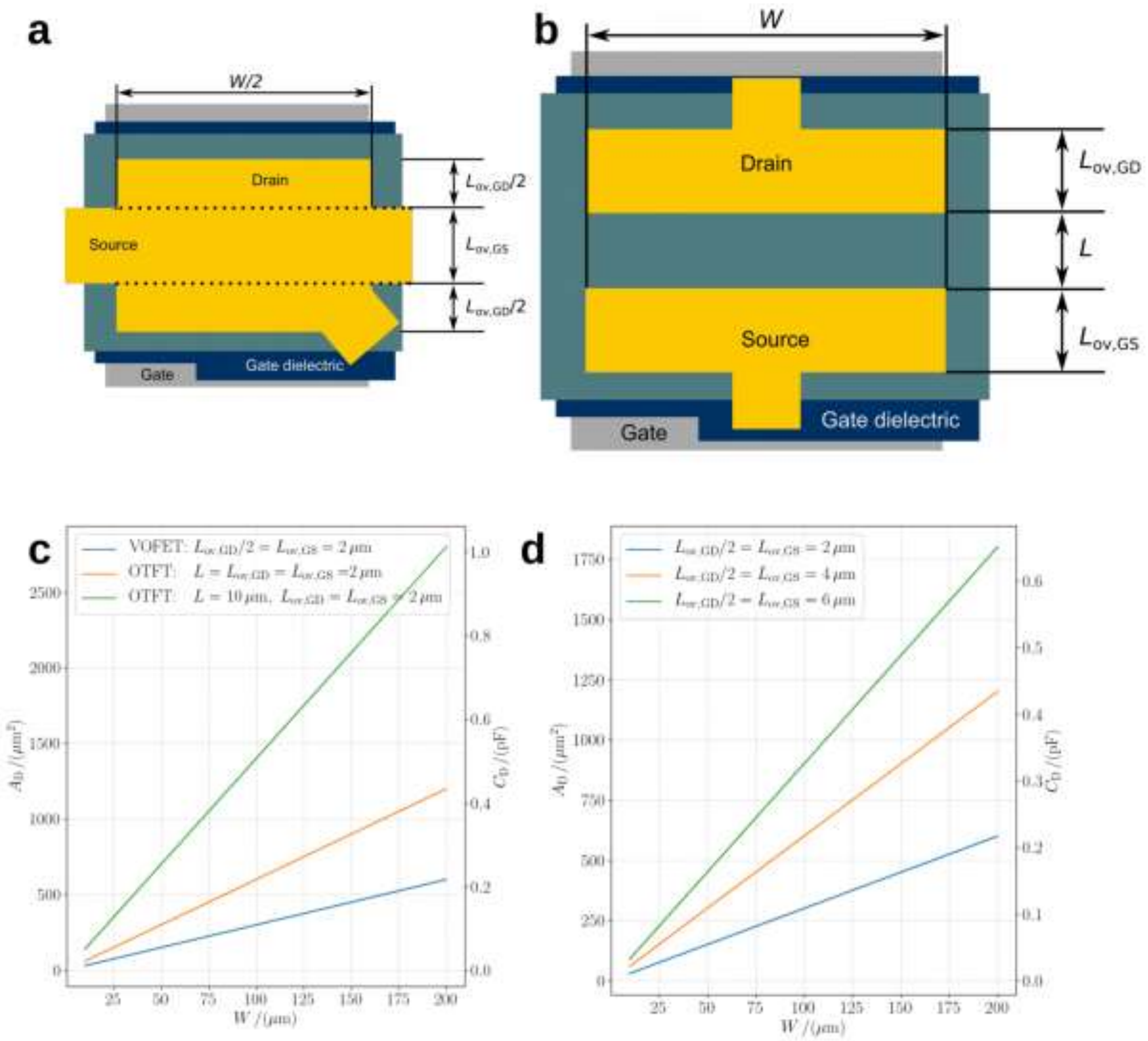

Figure S5: a Schematic of the VOFET geometry with the corresponding names of relevant metrics, and **b** an analogous schematic of the OTFT geometry (both devices are drawn to have the same W). Calculated areas A and capacitances C for variations of these geometry parameters comparing **c** OTFTs and VOFETs and **d** three different VOFET geometries. The gate dielectric was chosen to be Al_2O_3 with a thickness of 35 nm followed by a layer of CYTOP™ with a thickness of 40 nm

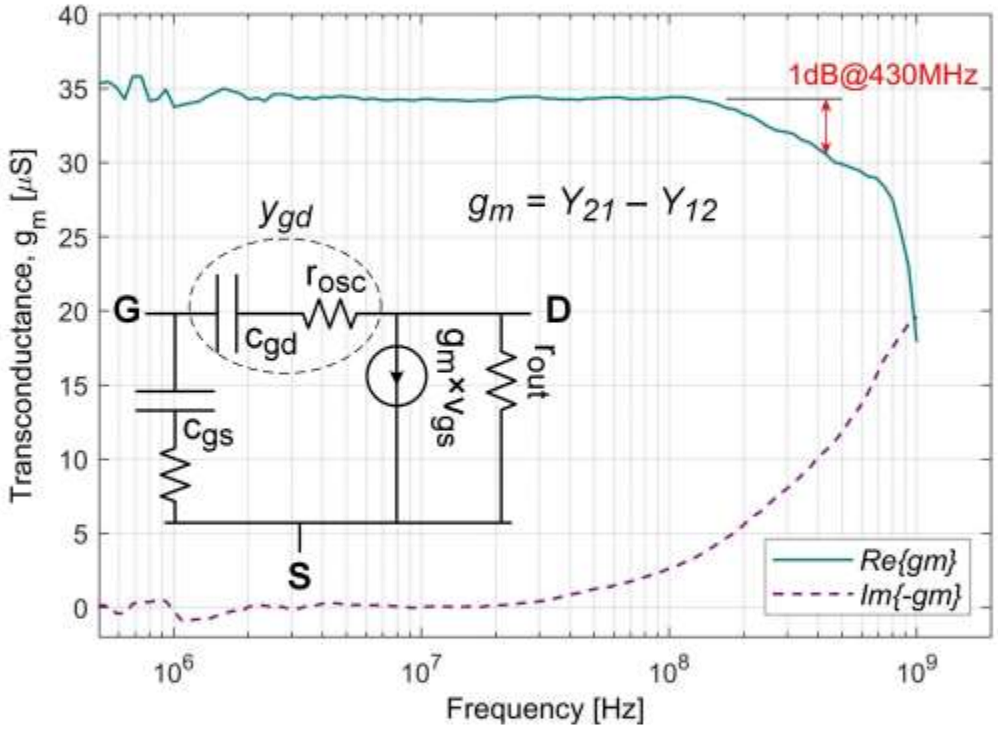

Figure S6: Extracted channel transconductance as a function of frequency at $I_D = -201$ µA, $V_{DS} = -9.8$ V for a DNTT-based VOFET.

References

[16] J. W. Borchert, R. T. Weitz, S. Ludwigs, and H. Klauk, "A critical outlook for the pursuit of lower contact resistance in organic transistors,"Advanced Materials **34**, 2, p. 2104075, 2022.

[23] B. Kheradmand-Boroujeni, M. P. Klinger, A. Fischer, H. Kleemann, K. Leo, and F. Ellinger, "A Pulse-Biasing Small-Signal Measurement Technique Enabling 40 MHz Operation of Vertical Organic Transistors, " Scientific Reports **8**, 7643, 2018.

[33] H. Kleemann, G. Schwartz, S. Zott, M. Baumann, and M. Furno, "Megahertz operation of vertical organic transistors for ultra-high resolution active-matrix display,"Flexible and Printed Electronics **5**, 1, 014009, 2020.

[38] U. Zschieschang, F. Ante, D. Kälblein, T. Yamamoto, K. Takimiya, H. Kuwabara, M. Ikeda, T. Sekitani, T. Someya, J.B. Nimoth, and H. Klauk, "Dinaphtho[2,3-b:2,3-f]thieno[3,2-b]thiophene (DNTT) thin-film transistors with improved performance and stability," Organic Electronics: physics, materials, applications **12**, 1370–1375, 2011.

[39] T. Yamamoto and K. Takimiya, "Facile synthesis of highly π-extended heteroarenes, dinaphtho[2,3-b: 2,3-f]chalcogenopheno[3,2-b]chalcogenophenes, and their application to field-effect transistors," Journal of the American Chemical Society **129**, 8, 2224–2225, 2007.

[40] S. Shinamura, I. Osaka, E. Miyazaki, and K. Takimiya, "Air-stable and high-mobility organic semiconductors based on heteroarenes for field-effect transistors," Heterocycles **83**, 6, 1187–1204, 2011.

[41] K. Takimiya, Y. Kunugi, Y. Konda, N. Niihara, and T. Otsubo, "2,6-Diphenylbenzo[1,2-b:4,5-b]dichalcogenophenes: A New Class of High-Performance Semiconductors for Organic Field-Effect Transistors," Journal of the American Chemical Society **126**, 16, 5084–5085, 2004.

[42] Y. Karpov, T. Erdmann, M. Stamm, U. Lappan, O. Guskova, M. Malanin, I. Raguzin, T. Beryozkina, V. Bakulev, F. Günther, S. Gemming, G. Seifert, M. Hambsch, S. Mannsfeld, B. Voit, and A. Kiriy, "Molecular Doping of a High MobilityDiketopyrrolopyrrole-Dithienylthieno[3,2-b]thiophene Donor-Acceptor Copolymer with F6-TCNNQ," Macromolecules **50**, 3, 914–926, 2017.

[43] F. Zhang and A. Kahn, “Investigation of the high electron affinity molecular dopant F6-TCNNQ for hole-transport materials,”Advanced Functional Materials **28**, 1, 1–8, 2018.

[44] R. Meerheim, S. Olthof, M. Hermenau, S. Scholz, A. Petrich, N. Tessler, O. Solomeshch, B. Lüssem, M. Riede, and K. Leo, “Investigation of C60F36 as low-volatility p-dopant in organic optoelectronic devices,” Journal of Applied Physics **109**, 10, 2011.

[45] K. Takimiya, T. Yamamoto, H. Ebata, and T. Izawa, “Design strategy for air-stable organic semiconductors applicable to high-performance field-effect transistors,” Science and Technology of Advanced Materials **8**, 273–276, 2007.

[46] H. Yagi, T. Miyazaki, Y. Tokumoto, Y. Aoki, M. Zenki, T. Zaima, S. Okita, T. Yamamoto, E. Miyazaki, K. Takimiya, and S. Hino, “Ultraviolet photoelectron spectra of 2,7-diphenyl[1]benzothieno[3,2-b][1] benzothiophene and dinaphtho[2,3-b:2,3-f]thieno[3,2-b]thiophene,” Chemical Physics Letters **563**, 55–57, 2013.

[47] J. Casado, M. M. Oliva, M. C. Deleado, R. P. Ortiz, J. J. Quirante, J. T. Navarrete, K. Takimiya, and T. Otsubo, “Hybrid organic semiconductors including chalcogen atoms in π-conjugated skeletons. Tuning of optical, redox, and vibrational properties by heavy atom conjugation,” Journal of Physical Chemistry A, vol. 110, no. 23, pp. 7422–7430, 2006.

[48] H. Kleemann, A. A. Zakhidov, M. Anderson, T. Menke, K. Leo, and B. Lüssem, “Direct structuring of C60 thin film transistors by photo-lithography under ambient conditions,” Organic Electronics **13**, 506–513, 2012.

[49] F. Dollinger, H. Iseke, E. Guo, A. Fischer, H. Kleemann, and K. Leo, “Electrically stable organic permeable base transistors for display applications”, Advanced Electronic Materials 5, 1900576 (2019).
